# High excess-free-fructose beverage consumption is not associated with prevalent allergy in US adults: a population-based analysis of NHANES 2005–2006

**DOI:** 10.1186/s41043-023-00439-6

**Published:** 2023-08-30

**Authors:** Ruili Yu, Lili Cai, Bo Yang

**Affiliations:** 1 Department of Allergy, Beijing Shijitan Hospital, Capital Medical University, Beijing, 100038 China; 2 Department of Laboratory Medicine, The Second Medical Center and National Clinical Research Center for Geriatric Diseases, Chinese PLA General Hospital, 28 Fuxing Road, Beijing, 100853 China; 3 Department of Hematology, The Second Medical Center and National Clinical Research Center for Geriatric Diseases, Chinese PLA General Hospital, 28 Fuxing Road, Beijing, 100853 China

**Keywords:** Allergic sensitization, Allergic symptoms, High excess-free-fructose (EFF) beverage, High-fructose corn syrup (HFCS), National Health and Nutrition Examination Survey (NHANES)

## Abstract

**Background:**

A strong association exists between high-excess free fructose (EFF) beverage consumption and prevalent allergy in children and adolescents; however, whether this association exists in the adult population is unclear. Therefore, this study aimed to investigate the relationship between high-EFF beverage intake and prevalent allergy.

**Methods:**

This cross-sectional study extracted data from the National Health and Nutrition Examination Survey 2005–2006. Adults aged ≥ 20 were eligible for inclusion, excluding those without complete information on beverage intake, allergic symptom survey, and allergen-specific immunoglobulin E test results. A total of 2077 adults were included. Univariate and multivariable logistic regression analyses determined the associations between high-EFF beverage consumption, prevalent allergic symptoms, and allergic sensitization.

**Results:**

After adjusting for confounders, there were no significant associations between high intake (vs. low) of sum of high-EFF beverage (adjusted odds ratio [aOR] = 1.10, 95% confidence interval [CI] 0.77, 1.57), apple juice (aOR = 0.95, 95% 0.55, 1.65), fruit drinks (aOR = 0.95, 95%CI 0.70, 1.29), soft drinks (aOR = 1.17, 95%CI 0.89, 1.55) and presence of allergic sensitization, or allergic symptoms. Stratified analyses also revealed no associations between high intake of high-EFF beverage in sum, presence of allergic symptoms or sensitization among individuals aged 20–39y, 40–59y, and ≥ 60y.

**Conclusions:**

Our findings indicate no independent association between frequent intake of high-EFF beverage and increased likelihood of allergy in US adults.

## Background

Preliminary epidemiological evidence indicates an association between the intake of high excess-free-fructose (EFF) beverages, including high-fructose corn syrup (HFCS) sweetened sodas, soft drinks, and fruit-flavored drinks and apple juice, and the prevalence of respiratory disease, especially asthma, in the USA. The association may be due to the high fructose-to-glucose ratio in these beverages and underlying fructose malabsorption [1–3]. Recent studies suggest that, in addition to childhood and adult asthma, high-EFF beverage is also connected to other harmful health effects such as glucose intolerance, chronic bronchitis, coronary heart disease [4–7], and idiopathic osteoarthritis [8].

Allergy is a damaging immune response by the body against a substance to which the body has become hypersensitive [9, 10]. The prevalence of allergic diseases, including asthma, allergic rhinitis, and atopic dermatitis, has increased to 40% in the global population [11, 12]. Our previous study demonstrated strong associations between more frequent high-EFF beverage consumption and a greater likelihood of allergic symptoms attacks, including eczema and allergic rhinitis, as well as allergic sensitization in children and adolescents [13]. However, this relationship has not yet been assessed in the adult population. Given that allergic illness also significantly negatively influences health in the overall adult population [14–16], this issue merits attention. Therefore, this study aimed to determine the relationship between high-EFF beverage intake, including HFCS-sweetened soft drinks, fruit drinks, and apple juice, and allergy in the general adult population, using a nationally representative database of the USA. We hypothesized that more frequent high-EFF beverage intake would be associated with an elevated probability of presence of allergic symptoms and allergic sensitization, independent of asthma.

## Methods and materials

### Study design and data source

In this cross-sectional, population-based investigation, data from the National Health and Nutrition Examination Survey (NHANES) database for the years 2005–2006 were analyzed. The Centers for Disease Control and Prevention (CDC) and the National Center for Health Statistics (NCHS) in the USA gathered the data. (http://www.cdc.gov/nchs/nhanes/). The NHANES survey, assessing the health status and nutritional conditions of the US’ adults, adolescents, and children. It employs a multistage, complex design to collect data that accurately represents the non-institutionalized population in the entire nation. The NCHS grants permission to researchers to use the data, which are made available for research purposes. Participants underwent a comprehensive evaluation, including a household interview and examinations in a mobile examination center (MEC), which entails laboratory tests, specialized measurements, and physical examinations. Consequently, evaluating subjects in the dataset provides a reliable and comprehensive assessment that reflects the characteristics of the entire population. [17] The Food Frequency Questionnaire (FFQ) was utilized by NHANES investigators to document people's eating habits during in-person interviews.

### Statement of ethics

The NHANES study underwent review and approval by the NCHS Research Ethics Review Board, and all participants in the survey provided informed consent. As a result, no additional ethical approval or informed permission were necessary for the secondary analyses performed. The NCHS approval can be accessed on the NHANES website (cdc.gov/nchs/nhanes/irb). Additionally, all NHANES data have undergone a process of de-identification to ensure anonymity.

### Study inclusion and exclusion

From the NHANES study cycle 2005–2006, all data were extracted for the current investigation. This particular data cycle was selected because it uniquely included survey questionnaires regarding participants’ allergic symptoms and a set of allergen-specific serum immunoglobulin E (IgE) tests for various allergens. The inclusion criteria for this study were adults ≥ 20 years of age. The exclusion criteria were subjects without complete information on high-EFF beverage consumption, IgE test, or subjects who did not answer the survey questionnaire regarding allergic symptoms.

### Variables of the study

#### High-EFF beverages consumption

For the present study, information was extracted on individuals’ frequency of consumption of high-EFF beverages, including (non-diet) fruit drinks, (non-diet) HFCS-sweetened soft drinks, and apple juice during the past year, from the FFQ. Individual values for the summer and the rest of the year were added to establish the average daily frequency of soft drink consumption. The NHANES specialist software (Diet*Calc, National Cancer Institute) assigns frequencies to FFQ responses using algorithms. The following algorithm, published by DeChristopher et al. [1], was used to examine the combined impact of all high-EFF beverages: Apple juice, fruit drinks (excluding 100% orange juice because it does not belong to high-EFF beverages), and soft drinks were assigned values of 0.0 for one or fewer times per month, 0.117 for 2–3 times per month, 0.357 for 1–4 times per week, and 1.0 for five or more times per week. The average daily intake of high-EFF beverages was calculated using these figures. For statistical purposes, the frequency categories of one or less times per month and 2–3 times per month were combined into a single group, yielding three frequency groups: low (0–3 times/month), medium (1–4 times/week), and high (5 times/week).

#### Assessment of allergic symptoms

To identify the existence of allergic symptoms, personal household interview data about allergic symptoms throughout the previous year were gathered from the NHANES allergy component files. Individuals who replied 'yes' to any of the following questions were considered to be allergic:

1. “During the past 12 months, have you had any allergy symptoms or an allergy attack?”
2. “During the past 12 months, have you had a problem with sneezing, runny, or blocked nose when you did not have a cold or the flu?”
3. “During the past 12 months, did a doctor or other health professionals tell you that you have a sinus infection?”
4. “Have you ever had an itchy rash that was coming and going for at least 6 months?” And, “Have you had this itchy rash at any time in the last 12 months?”
5. “During the past 12 months, have you had an episode of hay fever?”

#### Assessment of allergic sensitization

Individuals’ allergen-specific immunoglobulin E (IgE) was assessed using the ImmunoCAP 1000 System (Pharmacia Diagnostics, Freiburg, Germany). (cdc.gov/Nchs/Nhanes/2005-2006/AL_IGE_D.htm). Allergic sensitization was defined as a positive specific IgE response (≥ 0.35 kU/L) to at least one of the allergens tested [18].

### Other variables of the study

In-person interviews were conducted by trained interviewers using the Family and Sample Person Demographics questionnaires and the Computer-Assisted Personal Interviewing (CAPI) system (Confirmit Corp. New York, NY, USA) to obtain demographic data such as age, gender, race, family income-to-poverty ratio, and education level. The NHANES procedure was used to weight the data. The data were weighted under the guidance of the NHANES protocol.

The NHANES examination measurements were used to compute body mass index (BMI), which is calculated as body weight (kilograms) divided by height (meters squared). An electronic load cell scale was used to determine body weight, and a fixed stadiometer was used to determine standing height.

Total energy intake (kcal/day) of the participants was derived from the NHANES dietary interview and estimated from the 24-h dietary questionnaire.

Participants were categorized into non-smoker, former smoker, or current smoker based on the following criteria: lifetime smoking > 100 cigarettes and responded “yes” to the question “Do you smoke now?”, current smoker; lifetime smoking > 100 cigarettes but not currently a smoker, former smoker; and finally, lifetime smoking of fewer than 100 cigarettes, non-smoker.

Alcohol intake was classified based on survey responses: excessive alcohol consumption was defined as more than 21 standard drinks per week for men and more than 14 for women.

Physical activity levels were measured using the metabolic equivalent of tasks (METs)-min index, calculated from the product of weekly time spent in each activity reported by the participant multiplied by the MET value [19]. One MET equals one kcal/kg body weight per hour of energy expenditure. The individuals were further divided into three groups based on their MET values: ideal (500 MET-min per week), non-ideal (0–500 MET-min per week), and average (0–500 MET-min per week) [20].

Hyperlipidemia was defined as a “yes” answer to the following question: “To lower your blood cholesterol, have you ever been told by a doctor or other health professional to take prescribed medicine?”; or as total cholesterol > 240 mg/dL, an HDL-c level < 40 mg/dL, an LDL-c level ≥ 140 mg/dL or a triglyceride level ≥ 150 mg/dL.

Those who answered “yes” to the following questions were classified as having hypertension: “Were you told on 2 or more different visits that you had hypertension, also called high blood pressure?” or “Because of your (high blood pressure/hypertension), have you ever been told to... take prescribed medicine?”; or with an average of three consecutive measures of systolic blood pressure ≥ 140 mmHg; or with an average of three successive measurements of diastolic blood pressure ≥ 90 mmHg.

The following inquiries or tests were used to identify participants with diabetes mellitus (DM): a positive response to the question “Did a doctor tell you, you have diabetes?” “Do you take pills to lower blood sugar?” “Are you taking insulin?” or an HbA1c ≥ 6.5%, fasting glucose ≥ 126 mg/dL, or a glucose level ≥ 200 mg/dL in oral glucose tolerance test (OGTT) in the NHANES laboratory data [21].

Individuals with a family history of asthma, personal asthma history, and current asthma were identified by responding "yes" to the following question: “Including living and deceased, were any of your biological that is, blood relatives including grandparents, parents, brothers, sisters ever told by a health professional that they had...asthma?”, “Has a doctor or other health professional ever told you that you have asthma?” and “Do you still have asthma?”.

Mildew or musty smell in the home was identified through the questions: “In the past 12 months, has your home had a mildew odor or musty smell?” In addition, pet ownership in the past year was identified by the question: “Do any dogs, cats or other small furry animals, such as a rabbit, guinea pig or hamster, live or spend time in your home?” from the interview data of NHANES.

### Statistical analysis

NHANES uses a complex, multistage, probability sampling design to assure the accuracy of national estimates from the sample, wherein sampling weights (WTDRD1), pseudo-stratum (SDMVSTRA) and pseudo-cluster (SDMVPSU) provided by NHANES were applied in all analyses as guided by the NCHS. Continuous variables are presented as weighted means and standard errors (SE), while categorical variables are reported as unweighted numbers and weighted proportions. The SURVEYLOGISTIC statement was used to construct a logistic regression to examine the relationship between consumption of high-EFF beverages and allergic symptoms and sensitization. Covariates that displayed significant differences in the univariable regression analysis were included as adjustments in the multivariable models. Additionally, analyses that were stratified by age (20–39 years, 40–59 years, and 60 years) were carried out. Statistical significance was established at a two-sided P-value less than 0.05. All statistical analyses were performed using SAS statistical software (version 9.4, SAS Inc., Cary, NC, USA).

## Results

### Study population

**Fig. 1:**
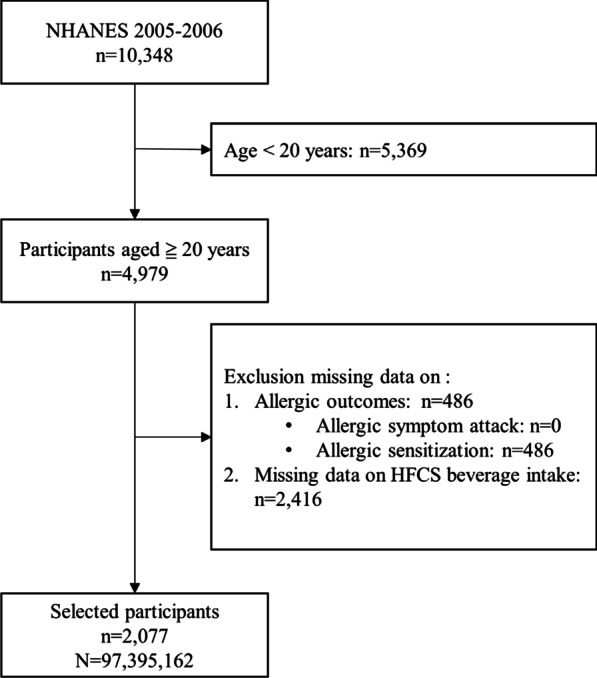
Flow diagram of study participants selection

Data of 10,348 participants from the NHANES 2005–2006 study cycle were extracted, of whom 4979 adults aged ≥ 20 years were eligible for inclusion. After excluding individuals without complete data on allergy and intake of high-EFF beverages, 2077 subjects remained for subsequent analyses. Using the sample weights provided by the NHANES database, this study sample size could be extrapolated back to 97,395,162 residents in the USA. The flow diagram of the study participant selection process is shown in Fig. 1.

### Sociodemographic characteristics, lifestyle, and medical history of the study population

The mean age of the study population was 46.7 ± 0.8 years. Most participants were non-Hispanic Whites (73.0%), not poor (89.1%), and never-smokers (52.8%). About 47.6% (988/2077) of the study population had at least one allergic symptom attack during the past 12 months, and 46.8% (971/2077) had objectively measured allergic sensitization. More than half of the subjects (58.0%) consumed high-EFF beverages in total at a high frequency (≥ 5 times/week) (Table 1).

**Table 1:** High-EFF beverage intake frequency, demographic, lifestyle and other characteristics of the general US adults, categorized by presence of allergic symptoms and allergic sensitization or not

Variables	Total	Allergic symptoms			Allergic sensitization		
	*N* = 97,395,162	Yes	No	*p* Value	Yes	No	*p* Value
	*n* = 2077	*n* = 988	*n* = 1089		*n* = 971	*n* = 1106	
*Sum of high-EFF beverage*				0.374			0.080
Low	483 (30.3)	244 (31.8)	239 (28.8)		185 (27.2)	298 (32.8)	
Medium	235 (11.7)	121 (12.4)	114 (10.9)		105 (10.6)	130 (12.6)	
High	1359 (58.0)	623 (55.8)	736 (60.3)		681 (62.2)	678 (54.6)	
*Apple juice*				**0.034**			0.518
Low	1619 (83.8)	792 (86.1)	827 (81.2)		743 (82.4)	876 (84.8)	
Medium	326 (12.3)	141 (10.7)	185 (14.1)		158 (13.5)	168 (11.3)	
High	132 (3.9)	55 (3.3)	77 (4.7)		70 (4.1)	62 (3.8)	
*Fruit drink*				0.098			0.556
Low	1390 (71.9)	678 (73.7)	712 (69.9)		635 (70.8)	755 (72.8)	
Medium	391 (16.3)	171 (16.3)	220 (16.3)		186 (17.0)	205 (15.8)	
High	296 (11.7)	139 (9.9)	157 (13.7)		150 (12.2)	146 (11.4)	
*Soft drink*				0.110			**0.001**
Low	739 (42.3)	366 (42.6)	373 (41.9)		290 (37.3)	449 (46.2)	
Medium	279 (12.1)	144 (13.7)	135 (10.4)		139 (12.4)	140 (11.9)	
High	1059 (45.6)	478 (43.7)	581 (47.7)		542 (50.3)	517 (41.9)	
*Age, years*	46.7 ± 0.8	46.2 ± 0.9	47.2 ± 1.4	0.570	44.3 ± 0.7	48.6 ± 1.0	** < 0.001**
20–64	1619 (82.7)	773 (83.7)	846 (81.6)	0.371	800 (86.3)	819 (79.8)	**0.004**
≥ 65	458 (17.3)	215 (16.3)	243 (18.4)		171 (13.7)	287 (20.2)	
*Gender*				**0.040**			** < 0.001**
Male	952 (47.4)	418 (44.4)	534 (50.7)		476 (52.2)	476 (43.5)	
Female	1125 (52.6)	570 (55.6)	555 (49.3)		495 (47.8)	630 (56.5)	
*Race*				**0.009**			** < 0.001**
Non-Hispanic White	1058 (73.0)	567 (77.1)	491 (68.6)		435 (67.0)	623 (77.9)	
] panic Black	503 (12.5)	218 (10.5)	285 (14.7)		265 (15.1)	238 (10.5)	
Mexican American	379 (6.9)	144 (5.5)	235 (8.5)		192 (7.9)	187 (6.1)	
Other Hispanic	61 (2.4)	25 (2.0)	36 (2.8)		33 (2.9)	28 (1.9)	
Others	76 (5.1)	34 (4.8)	42 (5.5)		46 (7.1)	30 (3.6)	
*Poverty income ratio*				0.587			**0.001**
Not poor (> 1)	1668 (89.1)	811 (89.7)	857 (88.5)		767 (87.0)	901 (90.8)	
Poor (≤ 1)	328 (10.9)	142 (10.3)	186 (11.5)		167 (13.0)	161 (9.2)	
Missing	81	35	46		37	44	
*BMI, kg/m*^*2*^				0.929			0.502
Underweight (< 18.5)	36 (1.6)	17 (1.4)	19 (1.7)		17 (1.3)	19 (1.8)	
Normal (18.5–24.9)	565 (31.4)	262 (30.9)	303 (31.9)		254 (30.7)	311 (31.9)	
Overweight (25–29.9)	701 (33.2)	342 (33.2)	359 (33.2)		329 (34.5)	372 (32.1)	
Obese (≥ 30)	755 (33.9)	359 (34.6)	396 (33.2)		359 (33.5)	396 (34.2)	
Missing	20	8	12		12	8	
*Smoking*				0.238			**0.002**
Never	1142 (52.8)	517 (50.4)	625 (55.4)		580 (57.9)	562 (48.6)	
Former	502 (23.6)	260 (25.7)	242 (21.3)		202 (20.0)	300 (26.4)	
Current	433 (23.6)	211 (23.9)	222 (23.3)		189 (22.1)	244 (24.9)	
*Excessive alcohol consumption*	53 (3.5)	28 (3.7)	25 (3.3)	0.739	28 (4.0)	25 (3.1)	0.324
*Physical activity MET-min/week*				0.202			0.373
Ideal (≥ 500)	846 (66.8)	425 (70.1)	421 (63.0)		406 (65.2)	440 (68.2)	
Not ideal (0 to < 500)	482 (33.2)	230 (29.9)	252 (37.0)		229 (34.8)	253 (31.8)	
Missing	749	333	416		336	413	
*Energy intake (kcal/day)*	2214.7 ± 37.2	2194.9 ± 50.6	2236.7 ± 45.4	0.505	2256.7 ± 58.9	2181.3 ± 35.6	0.216
*Comorbidities/medical history*							
Hyperlipidemia	996 (46.3)	492 (50.4)	504 (41.9)	0.138	455 (49.5)	541 (43.9)	**0.002**
With family history of asthma	446 (21.5)	262 (25.0)	184 (17.6)	**0.009**	233 (23.4)	213 (20.0)	0.147
With asthma history	269 (12.7)	192 (18.7)	77 (6.1)	** < 0.001**	182 (18.1)	87 (8.5)	** < 0.001**
With current asthma	165 (7.3)	129 (11.3)	36 (3.0)	** < 0.001**	112 (11.2)	53 (4.2)	** < 0.001**
Mildew or musty smell in home	320 (15.7)	176 (18.0)	144 (13.0)	0.062	150 (15.2)	170 (16.0)	0.695
Pet ownership past year	942 (54.6)	502 (61.5)	440 (46.9)	** < 0.001**	428 (52.9)	514 (56.0)	0.153

*EFF* excess free fructose, *BMI* body mass index, *MET* metabolic equivalent task, *DM* diabetes mellitus, *US* United States, *NHANES* National Health and Nutrition Examination Survey

Continuous variables are presented as mean ± SE

Categorical variables are presented as unweighted counts (weighted percentage)

*p* Value < 0.05 is shown in bold

### Associations between high-EFF beverage consumption and allergic symptoms

Associations between allergic symptoms and intake frequency of apple juice, fruit drinks, soft drinks, or in sum are shown in Table 2. After adjusting for relevant confounders in the multivariable analysis, compared with those who consumed soft drinks at a low frequency, adults who consumed soft drinks (aOR = 1.37, 95%CI 1.01–1.85, *p* value = 0.042) at a medium frequency had a significantly greater likelihood for prevalent allergic symptoms. Compared to those who consumed high-EFF beverages at a low frequency, adults who consumed high-EFF beverages in sum (aOR = 0.95, 95%CI 0.65–1.37, *p* value = 0.752), apple juice (aOR = 0.72, 95% 0.36–1.42, *p* value = 0.313), fruit drinks (aOR = 0.73, 95%CI 0.46–1.18, *p* value = 0.186), or soft drinks (aOR = 0.98, 95%CI 0.73–1.32, *p* value = 0.907) at a high frequency did not have a significantly greater odds for prevalent allergic symptoms (Table 2).

**Table 2:** Associations between high-EFF beverage consumption and presence of allergic symptoms in general US adults

Type of high-EFF beverage	*n* (%)	Adjusted OR (95% CI)^a^				
		Low intake	Medium intake	*p* Value	High intake	*p* Value
Sum of high-EFF beverage	2077 (100.0)	Ref	1.10 (0.64–1.90)	0.716	0.95 (0.65–1.37)	0.752
Apple juice	2077 (100.0)	Ref	0.80 (0.54–1.17)	0.230	0.72 (0.36–1.42)	0.313
Fruit drink	2077 (100.0)	Ref	1.08 (0.79–1.48)	0.616	0.73 (0.46–1.18)	0.186
Soft drink	2077 (100.0)	Ref	**1.37 (1.01–1.85)**	**0.042**	0.98 (0.73–1.32)	0.907

*p* < 0.05 is presented in bold to indicate statistical significance

*EFF* excess free fructose, *OR* odds ratio, *CI* confidence interval, *ref* reference, *US* United States

^a^Adjusted for variables with a *p* < 0.05 in Table 1, including gender, race, with family history of asthma, with asthma history, with current asthma, and pet ownership past year

### Associations between high-EFF beverage consumption and allergic sensitization

Associations between allergic sensitization and intake frequency of apple juice, fruit drinks, soft drinks, or in sum are shown in Table 3. After adjustment for relevant confounders in multivariable analysis, compared to those with the least consumption, adults who consumed high-EFF beverages in sum (aOR = 1.10, 95%CI 0.77–1.57, *p* value = 0.595), apple juice (aOR = 0.95, 95% 0.55–1.65, *p* value = 0.944), fruit drinks (aOR = 0.95, 95%CI 0.70–1.29, *p* value = 0.742), or soft drinks (aOR = 1.17, 95%CI 0.89–1.55, *p* value = 0.244) at a high frequency did not have a significantly greater odds for allergic sensitization (Table 3).

**Table 3:** Associations between high-EFF beverages consumption frequency and prevalent allergic sensitization in general US adults

Types of high-EFF beverage	*n* (%)	Adjusted OR (95% CI)^a^				
		Low	Medium	*p* Value	High	*p* Value
Sum of high-EFF beverage	1996 (96.1)^b^	Ref	0.89 (0.49–1.62)	0.684	1.10 (0.77–1.57)	0.595
Apple juice	1996 (96.1)^b^	Ref	1.07 (0.71–1.61)	0.729	0.95 (0.55–1.65)	0.944
Fruit drink	1996 (96.1)^b^	Ref	1.00 (0.75–1.32)	0.991	0.95 (0.70–1.29)	0.742
Soft drink	1996 (96.1)^b^	Ref	1.08 (0.69–1.69)	0.726	1.17 (0.89–1.55)	0.244

*EFF* excess free fructose, *OR* odds ratio, *CI* confidence interval, *ref* reference, *US* United States

^a^Adjusted for variables with a *p* < 0.05 in Table 1, including age (continuous), gender, race, poverty income ratio, smoking, hyperlipidemia, with asthma history, and with current asthma

^b^Excluding subjects with missing information on covariates

### Associations between allergic symptoms and sum of high-EFF beverage consumption, stratified by age

Stratified associations between total high-EFF beverage consumption and allergic symptoms by age are summarized in Table 4. Compared to individuals who consumed high-EFF beverages in sum at a low frequency, those consuming at a high frequency showed no significantly higher odds for allergic symptoms across all age groups (Table 4).

**Table 4:** Stratified associations between sum of high-EFF beverage consumption and presence of allergic symptoms in general US adults by age

Frequency	Adjusted OR (95% CI)^a^					
	20–39 years		40–59 years		≥ 60 years	
	*n* = 810	*p* value	*n* = 631	*p* value	*n* = 636	*p* value
Low	Ref		Ref		Ref	
Medium	1.15 (0.37–3.57)	0.792	0.95 (0.43–2.07)	0.881	1.31 (0.63–2.71)	0.445
High	0.89 (0.52–1.53)	0.647	0.90 (0.52–1.55)	0.676	1.03 (0.77–1.38)	0.822

*EFF* excess free fructose, *OR* odds ratio, *CI* confidence interval, *ref* reference, *US* United States

^a^Adjusted for variables with a *p* < 0.05 in Table 1 (except for stratified covariates), including gender, race, with family history of asthma, asthma history, current asthma, and pet ownership past year

### Associations between allergic sensitization and sum of high-EFF beverage consumption, stratified by age

Stratified associations between sum of high-EFF beverage consumption and allergic sensitization stratified by age are summarized in Table 5. Compared with those who consumed high-EFF beverages in sum at a low frequency, individuals consuming at a high frequency showed no significantly greater odds for allergic sensitization in all age groups (Table 5).

**Table 5:** Stratified associations between sum of high-EFF beverage consumption and presence of allergic sensitization in the general US adults by age

Frequency	Adjusted OR (95% CI)^a^					
	20–39 years		40–59 years		≥ 60 years	
	*n* = 785^b^	*p* Value	*n* = 611^b^	*p* Value	*n* = 600^b^	*p* Value
Low	Ref		Ref		Ref	
Medium	0.92 (0.39–2.18)	0.834	1.23 (0.41–3.70)	0.701	0.72 (0.37–1.37)	0.290
High	1.21 (0.65–2.25)	0.523	1.59 (0.88–2.88)	0.116	0.57 (0.32–1.01)	0.053

*EFF* excess free fructose, *OR* odds ratio, *CI* confidence interval, *ref* reference, *US* United States

^a^Adjusted for variables with a *p* < 0.05 in Table 1 (except for stratified covariates), including gender, race, poverty income ratio, smoking, hyperlipidemia, with asthma history, and with current asthma

^b^Excluding subjects with missing information on covariates

## Discussion

The present study demonstrated that, unexpectedly, among US adults aged ≥ 20y, greater high-EFF beverage intake, including apple juice, fruit drinks, soft drinks, or in sum, were not significantly associated with prevalent allergic symptoms or allergic sensitization. This finding remained the same when stratified the study population into different age group.

Most natural foods have a 1:1 fructose-to-glucose ratio with minimal EFF. Specifically, EFF is fructose that occurs when the fructose-to-glucose ratio exceeds 1:1, as in HFCS and apple juice. According to previous reports, apple juice naturally contains a high (≥ 2:1) fructose-to-glucose percentage. It is thus recognized as an EFF beverage; in contrast, the 100% orange juice with an approximately 1:1 fructose-to-glucose ratio is not an EFF beverage [15].

A recent study demonstrated that, in the children population, higher consumption of 100% juice, soda/sports/fruit drinks, and any combination, was associated with more than two times higher asthma incidence in the US [3]. In US adults, higher intake of any combination of HFCS-sweetened soda, fruit drinks, and apple juice was significantly associated with progressively higher asthma risk, rising from 59% higher for moderate consumers to a plateau of 89% higher among those consuming 5–7 times/week versus those never/seldom drinking high-EFFs, independent of potential confounders such as age, sex, BMI, smoking, education level, and total energy intake [2]. In our prior research, which targeted the US population of children and adolescents, we looked at non-asthma allergic disorders. A higher frequency of high-EFF beverage intake was found to be independently associated with prevalent allergy [11]. Although the studies cited above suggest the links between the consumption of high-EFF beverages and asthma in children and adults and allergy in children and adolescents, the relationships between high-EFF beverage intake and adult allergy have yet to be investigated.

The “intestinal advanced glycation end-products (enFruAGE) hypothesis” indicates that fructose malabsorption due to regular intake of high-EFF and HFCS contributes to unpaired fructose reactivity in the gastrointestinal tract and intestinal in situ formation of pro-inflammatory enFruAGEs, which once absorbed, travels beyond the intestinal boundaries to other tissues. Then, these enFruAGEs may finally lead to increased IgE responses that correlate with stimulation [1].

Results of the present study show no significant associations between intake of high-EFF drinks and the risk of experiencing an allergic symptoms and allergic sensitization. These findings are inconsistent with our previous work highlighting the relationships between high-EFF drink consumption and allergy in kids and teenagers [11] and need further reasonable explanations.

First, because of the limited sample size in the sub-analysis, statistical power may have been insufficient. Another possible explanation would be the presence of so-called immune tolerance to allergens [22]. Immune tolerance refers to developing a long-term clinical tolerance towards allergens, whereby the immune system no longer reacts exaggeratedly to these substances. This phenomenon is characterized by specific changes in the immune response, particularly involving memory-type allergen-specific T and B cells, mast cells, and basophils. These alterations raise activation thresholds for these immune cells, preventing them from triggering allergic symptoms [22]. The concept of immune tolerance provides a partial explanation for the observation that associations between allergies and specific factors are more commonly found in children and adolescents than adults. During childhood and adolescence, the immune system is still developing and maturing. As a result, it is more susceptible to environmental influences and more prone to developing hypersensitivity reactions, such as allergies. On the other hand, the immune system of adults has undergone a more stable and established immune response pattern, making it less likely to exhibit allergic reactions to the same extent.

### Strengths and limitations

The present study is strengthened by the use of the NHANES database, which is comprehensive and nationally representative, drawn from a large and diverse sample of participants from the population of the USA. Therefore, the findings are likely generalizable to the overall US population. Nonetheless, the study has several limitations. First, the current report is of cross-sectional design, which does not allow causal relationships to be made. Second, the questionnaire was administered once to collect data on high-EFF beverage consumption and allergic symptoms. Thus, inaccurate reporting or recall bias may have occurred. Third, non-specific allergic symptoms, lack of control of all the participants’ underlying chronic illnesses, and uncollected environmental factors may bias the results. Fourth, caution should be taken when generalizing the US findings to other countries since the use of HFCS in soft drinks may differ. Fifth, the study is constrained by the utilization of data spanning back 16 years, lacking recent epidemiological insights into the dietary landscape of general US adults. Sixth, using frequency as the sole criterion for assessment may not accurately determine the actual quantity of high-EFF beverages consumed.

## Conclusions

Unlike that in children and adolescents, no consistent and significant associations were found between frequent intake of high-EFF beverages, presence allergic symptoms, and allergic sensitization in US adults 20–79 y. Future longitudinal studies may help to provide further evidence to support our findings.

## Data Availability

The datasets analyzed during the current study are available from the corresponding author on reasonable request.

## Abbreviations

EFF: High-excess free fructose
IgE: Immunoglobulin E
aOR: Adjusted odds ratio
CI: Confidence interval
HFCS: High-fructose corn syrup
NHANES: National Health and Nutrition Examination Survey
MEC: Mobile examination center
